# Anti-inflammatory duration of action of fluticasone furoate/vilanterol trifenatate in asthma: a cross-over randomised controlled trial

**DOI:** 10.1186/s12931-018-0836-6

**Published:** 2018-07-13

**Authors:** George Bardsley, Peter Daley-Yates, Amanda Baines, Rodger Kempsford, Mathew Williams, Tony Mallon, Irene Braithwaite, Kylie Riddell, Shashidhar Joshi, Philippe Bareille, Richard Beasley, James Fingleton, on behalf of the study team

**Affiliations:** 1 Medical Research Institute of New Zealand, Private Bag 7902, Newtown, Wellington, 6242 New Zealand; 2 Respiratory Clinical Development, GlaxoSmithKline Research and Development, Stockley Park, Uxbridge, UK; 3 Medicines Development Centre, GlaxoSmithKline Research and Development, Stevenage, UK; 4 GlaxoSmithKline Research and Development, 82 Hughes Ave, Ermington, NSW 2115 Australia; 5 Quantitative sciences, GlaxoSmithKline, Bangalore, India

**Keywords:** Asthma, Nitric oxide, Clinical trial

## Abstract

**Background:**

Fluticasone furoate/Vilanterol trifenatate (FF/VI) is an inhaled corticosteroid/long-acting beta-agonist combination with a prolonged bronchodilator duration of action. We characterised the time-course of onset and offset of airway anti-inflammatory action of FF/VI, as assessed by fraction of exhaled nitric oxide (FeNO), and compared this to the bronchodilator duration of action.

**Methods:**

A single-centre, randomised, double-blind, placebo-controlled, two-period, crossover study was undertaken in 28 steroid-naïve adults with asthma. Participants with an FEV_1_ ≥ 60% predicted, reversible airway disease, and FeNO > 40 ppb received FF/VI 100/25 mcg or placebo once daily for 14 days. FeNO and peak expiratory flow were measured twice-daily during treatment and during a 21-day washout period. FEV_1_ was measured for five days from treatment cessation. The primary outcome measure was FeNO change from baseline ratio for 21 days following treatment cessation.

**Results:**

In the 27 subjects who completed the study, median (range) baseline FeNO was 87 ppb (42–212). FF/VI 100/25 mcg reduced FeNO by day 3, ratio FF/VI versus placebo 0.72 (95% confidence interval 0.61–0.86) with the maximum reduction occurring at day 14, 0.32 (0.27–0.37). Following cessation of treatment FeNO remained suppressed for 18 days, ratio on day 18 0.77 (0.59–1.00), whereas improvements in FEV_1_ and peak flow were maintained for 3 to 4 days post-treatment.

**Conclusions:**

The anti-inflammatory duration of action of FF/VI is consistent with the high glucocorticoid receptor affinity and long lung retention of fluticasone furoate. The anti-inflammatory effect of FF/VI was of greater duration than its bronchodilator effect in adults with mild asthma.

Funding GlaxoSmithKline (201499).

**Trial registration:**

Prospectively registered on ClinicalTrials.gov registry number NCT02712047.

**Electronic supplementary material:**

The online version of this article (10.1186/s12931-018-0836-6) contains supplementary material, which is available to authorized users.

## Background

Combination inhaled corticosteroid (ICS) / long-acting beta-agonist (LABA) therapy forms the cornerstone of guideline recommended management of moderate to severe asthma [1] and in some countries ICS/LABA combination inhalers are the most commonly prescribed ICS containing medication [2]. Most ICS/LABA medications require twice-daily dosing, however adherence to ICS-containing therapy is low and changing inhaled therapy to a once-daily regimen is suggested by international guidelines as a strategy for improving adherence [1, 3]. These findings have led to the development of ICS and LABA medications with a prolonged duration of action, allowing once-daily dosing.

Fluticasone furoate / Vilanterol trifenatate (FF/VI) 100/25 mcg is an ICS/LABA combination licensed for the once-daily treatment of asthma and chronic obstructive pulmonary disease [4]. In people with asthma, after a single dose of FF/VI, bronchodilation was maintained for 72 h post dose with clinically significant bronchodilation persisting for at least 48 h [5]. However, the onset and duration of anti-inflammatory action of FF/VI has not been studied and since LABA monotherapy without concomitant ICS therapy is associated with increased mortality in asthma [6], it is important to determine whether the anti-inflammatory duration of action of FF/VI is of at least the same duration as the bronchodilator activity. Although this is of limited relevance for chronic therapy in treatment adherent patients it may be important for patients who miss one or more doses. The primary objective of this study was to characterise the duration of anti-inflammatory activity of FF/VI administered to patients with asthma. We hypothesised that FF would exhibit a prolonged duration of anti-inflammatory action, as the FF component is an ICS with higher glucocorticoid receptor affinity [7] and more prolonged lung retention [8], compared to other ICS.

## Methods

### Trial design and participants

This study was a single-centre, randomised, double-blind, placebo-controlled, two-period, crossover, repeat dose study carried out by the Medical Research Institute of New Zealand (MRINZ) in Wellington, New Zealand.

Eligible participants were aged 18 to 65 inclusive, had a doctor’s diagnosis of asthma for at least six-months prior to enrolment, were prescribed a short-acting β2-agonist for at least 12 weeks prior to screening, had a fraction of exhaled nitric oxide (FeNO) of greater than 40 ppb at the time of screening, screening pre-bronchodilator forced expiratory volume in one second (FEV_1_) of at least 60% predicted, (NHANES III reference values) and demonstrated presence of reversible airways disease within the last six months, defined as an increase in FEV1 of at least 12% from baseline and an absolute change of at least 200 mL within the 30 min following inhalation of 400mcg salbutamol via a valved holding chamber.

Additional inclusion and the exclusion criteria are provided in the online supplement.

Prospective ethics approval was obtained from the Health and Disability Ethics Committees, New Zealand (Reference 16/STH/13). The trial was prospectively registered on the ClinicalTrials.gov registry (NCT02712047). Written informed consent was obtained prior to any study-specific procedures. The trial was performed in accordance with the ethical principles of the Declaration of Helsinki, International Conference on Harmonisation of Good Clinical Practice Guidelines, and applicable regulatory requirements.

### Randomisation and blinding

Participants were randomly assigned to one of two treatment sequences (AB or BA, where A is placebo and B is FF/VI 100/25mcg) in a 1:1 ratio in accordance with the randomization schedule generated prior to the start of the study by the study statistician, using validated GlaxoSmithKline (GSK) internal software. Participants, investigators, and the sponsor were blinded to sequence allocation for the duration of the study.

**Fig. 1:**
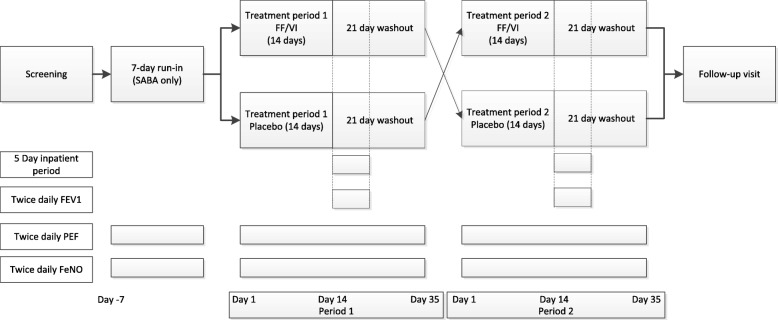
Study schematic. SABA, short-acting beta-agonist; FF/VI, fluticasone furoate / vilanterol; FeNO, fraction of exhaled nitric oxide; PEF, peak expiratory flow, FEV_1_, forced expiratory volume in one second

After randomisation, participants completed a 14-day treatment period, during which participants received inhaled study medication once-daily, followed by a 21-day, short-acting beta-agonist (SABA) only, washout period, before crossing over to the other arm of the study. A period of up to 28 days was permitted between study periods. The study schematic is shown in Fig. 1.

### Procedures

Eligible participants completed a 7 day run-in period prior to randomisation to confirm ability to perform FeNO and peak expiratory flow (PEF) measurements without supervision. Baseline FeNO (Niox Vero, Aerocrine, Sweden) and spirometry (Jaeger Masterscreen, Erich-Jaeger, Germany) measurements were completed in accordance with American Thoracic Society/ European Respiratory Society guidelines.

Patients were admitted into the Clinical Trials Unit at Wellington Hospital for a five day monitoring period, on the first day of which the final administration of study medication was witnessed by investigators. Participants completed twice daily PEF and FeNO measurements throughout the treatment and monitoring periods, as well as FEV_1_ measurements at each visit and twice daily during the inpatient period. Inpatient morning (AM) FEV_1_ readings were obtained between 7 am and 10 am, with evening (PM) readings 12 h after the AM reading. Each participant performed inpatient readings at a similar time each day and retained the same Niox Vero and PEF meter for the duration of the study, to minimise test to test variability. Participants avoided nitrate-rich foods for the duration of the study. As forced expiratory manoeuvres may cause short-term alteration in FeNO the measurement sequence was FeNO followed by FEV_1_ and then PEF, at all time-points. FEV_1_ and PEF measurements taken within 6 h of SABA use were not included in the analysis.

Blood eosinophil levels (XE platform, Sysmex Corporation, Japan) and serum periostin levels (see Additional file 1) were determined at baseline, at the end of treatment, and seven and 21 days after cessation of repeat dose treatment, for exploratory analyses. This study also included an exploratory biomarker investigation using serum samples collected at the same time as the blood eosinophil samples. The global metabolomics and lipidomic profiles were measured pre-dose and on days 15 and 21 in each period, and these exploratory analyses will be reported in detail in a separate manuscript.

### Outcomes

The primary end point was change from baseline FeNO over time following the cessation of repeat dose treatment with FF/VI. Pre-specified secondary end points were change from baseline FeNO over the FF/VI treatment period, measurement of PEF during treatment and following cessation of repeat dose treatment with FF/VI, and measurement of FEV_1_ pre-treatment and twice daily for five days after cessation of repeat dose treatment with FF/VI, with a final FEV_1_ reading on day seven.

### Statistical analysis

As a descriptive study, an estimation approach was adopted to address the primary objective. Point estimates and corresponding 95% confidence intervals (CI) were constructed for the treatment ratio of FF/VI 100/25mcg and placebo. 28 subjects were to be recruited to ensure 24 evaluable subjects. Based upon a within-subject coefficient of variation of 21.6%, estimated from a previous study with a sample size of 24 subjects [9], it was estimated that the lower and upper bounds of the 95% CI for the treatment ratio (FF/VI vs Placebo) for FeNO would be approximately 14% of the point estimate.

Following loge-transformation, the primary end point of FeNO change from baseline ratio following cessation of treatment was analysed using a mixed effects repeated measures model with fixed effect terms for period, treatment, day, time, (period-level baseline)*day*time interaction term, treatment*day*time interaction term, period-level baseline and subject-level baseline. Day*time interaction term was fitted as a repeated measure. FeNO was loge-transformed prior to deriving subject-level baseline and period-level baseline. Point estimates and their associated 95% CI were constructed for the difference [FF/VI 100/25mcg] – [Placebo] at each time point. The point estimates and their associated 95% CI were then back-transformed to provide point estimates and 95% CI for the ratios, [FF/VI 100/25mcg]/[Placebo], for each time point.

Secondary end points of FeNO change from baseline ratio during treatment and FEV_1_ were analysed similarly to the primary FeNO endpoint. FEV1 was not log-transformed. PEF, safety data, and biomarker data were summarised descriptively. As serum periostin levels and blood eosinophil levels show significant inter-subject variability, values were normalised to the individual’s baseline level and response to treatment was expressed as a percentage change from baseline (FF/VI minus placebo).

### Role of the funding source

The trial was conceived by the academic investigators and sponsored and funded by GlaxoSmithKline. The protocol, available as part of the online data supplement, was developed in collaboration between the principal academic investigators and employees of the sponsor. The first draft of this manuscript was prepared by the academic investigators without medical writing assistance.

## Results

The study was conducted between April 2016 and February 2017, during which 32 potential participants were screened. Four participants were excluded on the grounds of a screening FeNO ≤40 ppb and 28 participants were enrolled. One participant withdrew after screening but prior to taking any study medication. 27 participants successfully completed both study periods and were included in the final analysis (Additional file 1: Figure S1).

Baseline characteristics of the 27 randomised participants are shown in Table 1. Participants had mild to moderate airflow obstruction at baseline, mean (standard deviation) FEV_1_ percent predicted 87.7 (10.6). Median (range) baseline FeNO and blood eosinophils were 87 ppb (42 to 212) and 0.33 × 10^9/L (0.18 to 1.18) respectively.

**Table 1:** Baseline Characteristics of Randomised participants

Characteristic	Study population (*N* = 27)
Age (yrs.)	24.5 (8.1)
Sex	
Female	10 (37%)
Male	17 (63%)
Ethnic Origin	
Māori	4 (15%)
NZ European	20 (74%)
Other	3 (11%)
History of allergic rhinitis	14 (51.9%)
Body-mass index^a^	24.6 (3.5)
FEV_1_ before bronchodilation (% predicted)	87.7 (10.6)
Peak Expiratory Flow (l/min)	443 (99)
FeNO (ppb)	87 (42–212)
Blood eosinophil count (× 10^9/L)	0.33 (0.18–1.18)
Blood eosinophil count ≥0.27 × 10^9/L	24 (88.9%)
Serum Periostin (ng/ml)	187.5 (109.4–442.5)

Values are given as mean (SD), median (Range), or n (%). ^a^The body-mass index is the weight in kilograms divided by the square of the height in meters

**Fig. 2:**
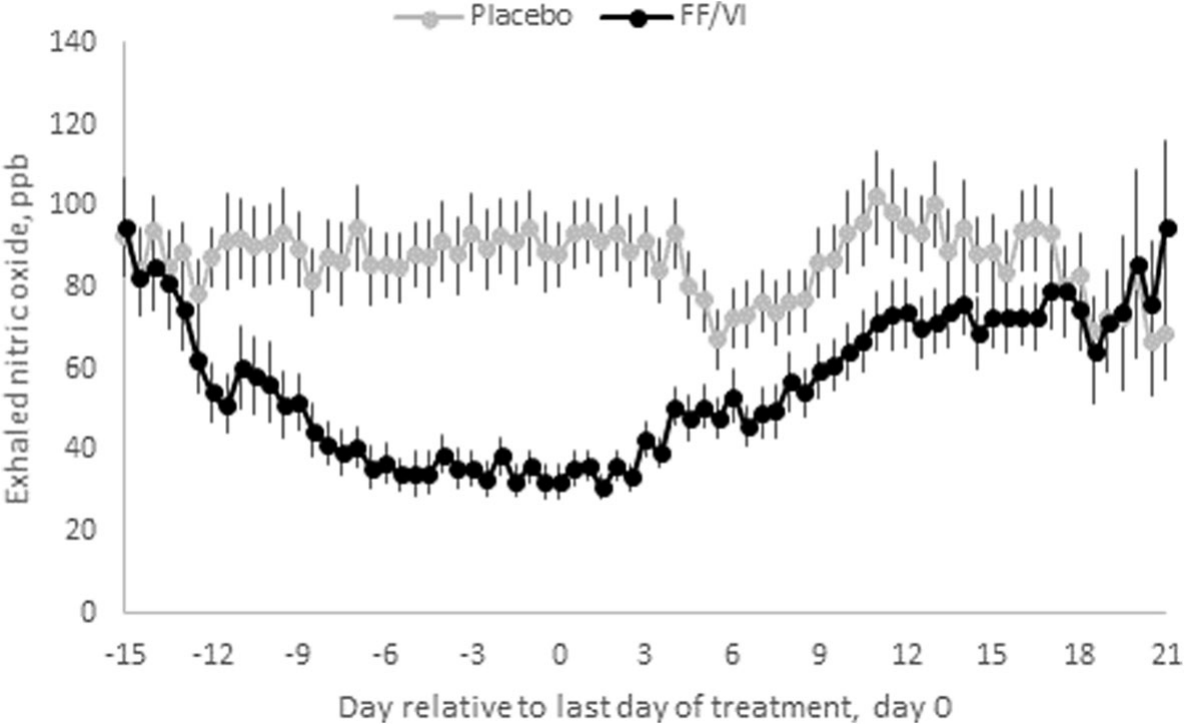
Time-course of anti-inflammatory activity of FF/VI. Mean exhaled nitric oxide (ppb), during treatment and after cessation of treatment, plotted over time, for placebo (grey line) and FF/VI (black line). Error bars denote the standard error

The time-course of FeNO during the 14-day treatment and 21-day washout periods is shown in Table 2 and Fig. 2. Summary data by treatment period are provided in Additional file 1: Table S2. Compared with placebo, FF/VI 100/25mcg treatment decreased the FeNO by the third day following onset of treatment, change from baseline ratio FF/VI versus placebo 0.72 (95% CI 0.61 to 0.86). The maximum reduction in FeNO occurred on day 14, change from baseline ratio FF/VI versus placebo 0.32 (0.27 to 0.37).

**Table 2:** FeNO change from baseline ratio following onset and cessation of treatment

A: FeNO change from baseline and FF/VI:placebo ratio following onset of treatment							
Day of treatment	Time point	Geometric LSmean				Ratio (FF/VI vs Placebo)	95% CI of the Ratio
		n	FF/VI 100/25 mcg	n	Placebo		
1	PM	25	0.97	26	0.90	1.09	(0.91,1.29)
2	AM	27	0.91	27	1.00	0.91	(0.77,1.07)
	PM	27	0.81	25	0.89	0.91	(0.77,1.08)
3	AM	27	0.75	27	0.95	0.79	(0.67,0.94)
	PM	27	0.64	25	0.88	0.72	(0.61,0.86)
4	AM	26	0.52	27	0.90	0.58	(0.49,0.69)
	PM	27	0.48	27	0.89	0.54	(0.46,0.64)
5	AM	27	0.46	27	0.99	0.47	(0.39,0.55)
	PM	26	0.45	26	0.95	0.47	(0.40,0.56)
6	AM	26	0.45	27	0.97	0.46	(0.39,0.55)
	PM	27	0.41	27	0.96	0.42	(0.36,0.50)
7	AM	27	0.45	26	0.96	0.47	(0.39,0.56)
	PM	27	0.39	27	0.88	0.45	(0.38,0.53)
8	AM	27	0.40	27	0.94	0.43	(0.37,0.51)
	PM	27	0.37	26	0.90	0.41	(0.34,0.48)
9	AM	26	0.39	27	0.99	0.40	(0.33,0.47)
	PM	25	0.34	25	0.89	0.38	(0.32,0.46)
10	AM	26	0.36	26	0.93	0.39	(0.33,0.46)
	PM	27	0.36	26	0.90	0.40	(0.34,0.47)
11	AM	27	0.33	27	0.92	0.36	(0.30,0.42)
	PM	27	0.31	27	0.88	0.36	(0.30,0.42)
12	AM	27	0.39	27	0.94	0.42	(0.35,0.49)
	PM	27	0.35	27	0.91	0.39	(0.33,0.46)
13	AM	24	0.34	25	0.99	0.35	(0.29,0.41)
	PM	24	0.32	25	0.94	0.34	(0.29,0.40)
14	AM	23	0.38	23	1.00	0.37	(0.32,0.44)
	PM	23	0.32	22	1.00	0.32	(0.27,0.37)
B: FeNO change from baseline and FF/VI:placebo ratio following cessation of treatment							
Day relative to last dose	Time point	Geometric LSmean				Ratio (FF/VI vs Placebo)	95% CI of the Ratio
		n	FF/VI 100/25 mcg	n	Placebo		
1	AM	27	0.38	27	1.03	0.36	(0.31,0.43)
	PM	27	0.32	27	0.98	0.33	(0.28, 0.39)
2	AM	27	0.38	27	0.99	0.38	(0.32, 0.45)
	PM	27	0.35	27	0.94	0.37	(0.31, 0.44)
3	AM	27	0.45	27	0.98	0.46	(0.39, 0.54)
	PM	27	0.42	27	0.91	0.47	(0.40, 0.56)
4	AM	27	0.55	27	0.98	0.56	(0.47, 0.66)
	PM	27	0.49	27	0.85	0.58	(0.49, 0.69)
5	AM	27	0.52	27	0.83	0.63	(0.53, 0.75)
	PM	26	0.48	26	0.72	0.67	(0.57, 0.80)
6	AM	26	0.53	26	0.79	0.67	(0.56, 0.80)
	PM	26	0.48	24	0.78	0.62	(0.52, 0.74)
7	AM	27	0.49	27	0.82	0.60	(0.51, 0.71)
	PM	25	0.49	25	0.79	0.62	(0.52, 0.74)
8	AM	27	0.56	25	0.82	0.69	(0.58, 0.81)
	PM	26	0.56	27	0.82	0.68	(0.57, 0.80)
9	AM	27	0.61	27	0.92	0.66	(0.56, 0.78)
	PM	27	0.62	27	0.91	0.68	(0.57, 0.80)
10	AM	26	0.66	24	1.01	0.66	(0.55, 0.78)
	PM	27	0.67	27	0.98	0.68	(0.58, 0.81)
11	AM	26	0.77	26	1.06	0.73	(0.61, 0.86)
	PM	27	0.74	27	1.01	0.73	(0.61, 0.86)
12	AM	27	0.73	27	1.01	0.72	(0.61, 0.85)
	PM	27	0.70	27	0.99	0.71	(0.60, 0.84)
13	AM	25	0.76	27	1.05	0.72	(0.61, 0.86)
	PM	25	0.74	25	0.95	0.78	(0.66, 0.93)
14	AM	27	0.79	26	1.00	0.79	(0.66, 0.93)
	PM	26	0.63	25	0.95	0.67	(0.56, 0.79)
15	AM	26	0.76	26	0.97	0.79	(0.66, 0.93)
	PM	25	0.73	25	0.92	0.79	(0.66, 0.94)
16	AM	27	0.75	26	1.01	0.74	(0.62, 0.87)
	PM	27	0.73	26	1.01	0.73	(0.61, 0.86)
17	AM	26	0.79	25	1.03	0.77	(0.65, 0.91)
	PM	16	0.79	18	0.97	0.81	(0.65, 1.01)
18	AM	14	0.75	17	0.98	0.77	(0.61, 0.96)
	PM	10	0.67	14	0.87	0.77	(0.59, 1.00)
19	AM	10	0.78	14	0.96	0.81	(0.63, 1.05)
	PM	6	0.67	12	0.93	0.72	(0.52, 1.00)
20	AM	6	0.77	12	0.99	0.78	(0.56, 1.09)
	PM	5	0.72	7	0.92	0.78	(0.53, 1.14)
21	AM	6	0.85	7	1.00	0.85	(0.59, 1.22)
	PM	5	0.75	7	0.99	0.75	(0.50, 1.13)

Legend: Summary of repeated measures statistical analysis of ratio from baseline exhaled nitric oxide (ppb) data after A) onset and B) cessation of repeat dose of treatment. Decrease in n value is seen from Day 17 after last dose due to permitted study visit windows

Following cessation of FF/VI 100/25mcg treatment the FeNO gradually increased towards the values following placebo, remaining suppressed until approximately day 18, ratio 0.77 (0.59 to 1.00). By inspection of Fig. 2 it can be seen that the rate of onset of FeNO suppression was greater than the rate of offset.

**Fig. 3:**
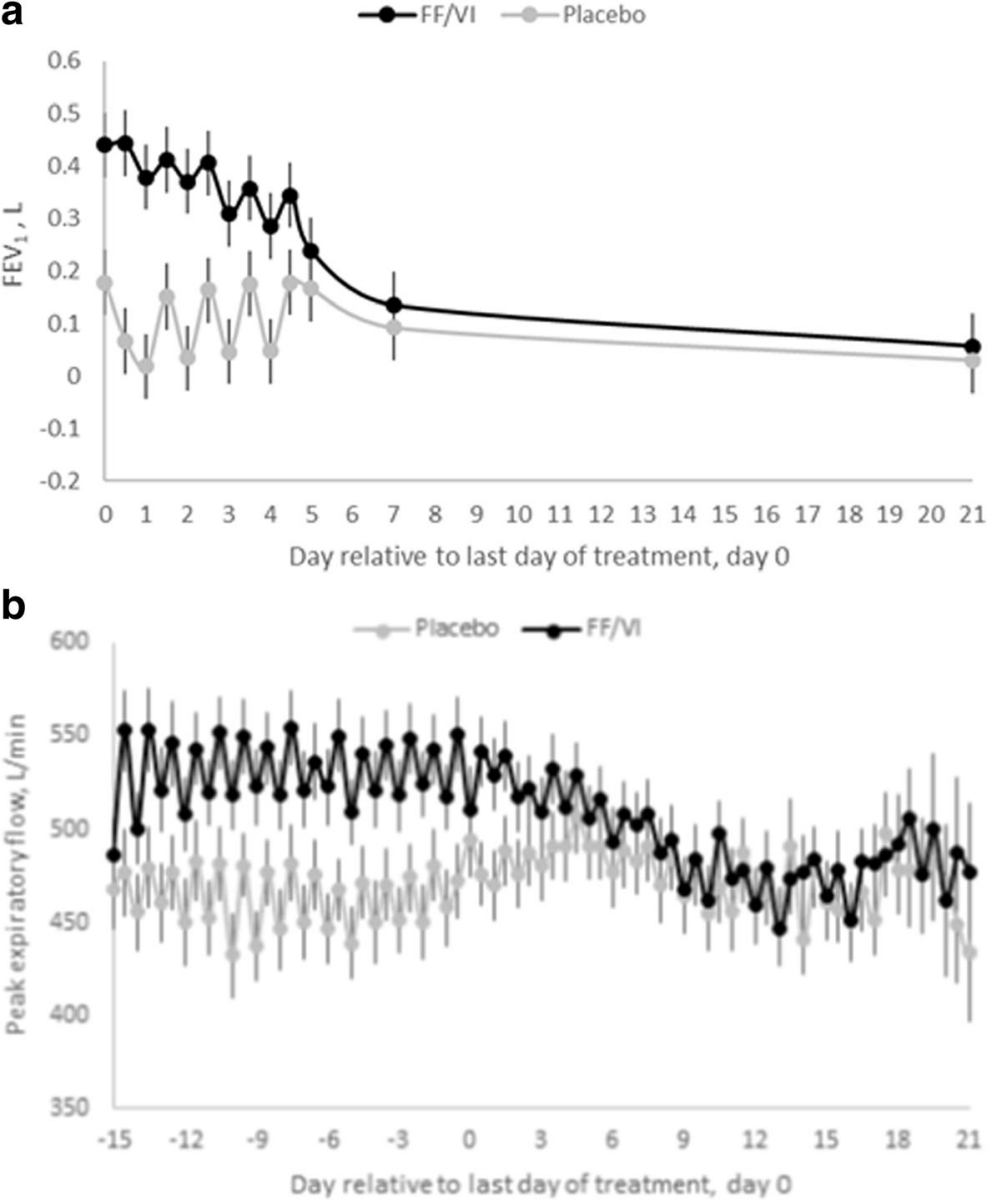
Time-course of bronchodilator activity of FF/VI. **a**: FEV_1_ change from baseline. **b**: PEF. Mean (**a**) FEV_1_ (L) after cessation of treatment and (**b**) Peak expiratory flow (L/min), during treatment and after cessation of treatment, plotted over time, for placebo (grey line) and FF/VI (black line). Error bars denote the standard error

FF/VI 100/25 mcg was associated with significant bronchodilation as measured by FEV_1_ (Table 3, Fig. 3a), adjusted mean FEV_1_ change from baseline compared with placebo 0.38 (0.28 to 0.48), 12 h after the last dose on day 14. After cessation of FF/VI treatment, FEV_1_ remained significantly different from placebo until the PM measurement on Day 4. The increase in FEV_1_ with FF/VI relative to placebo was greater than the minimum perceivable improvement (0.23 L) [10] until day 3 after cessation of treatment, difference 0.18 L (0.08, 0.28). PEF remained significantly greater than placebo until the AM measurement on Day 4 (Additional file 1: Table S1, Fig. 3b).

**Table 3:** FEV_1_ change from baseline and FF/VI vs placebo treatment difference following cessation of treatment

Day relative to last dose	Time point	Adjusted Mean FEV_1_				Difference (FF/VI vs Placebo)	95% CI of the difference
		n	FF/VI 100/25 mcg	n	Placebo		
0	AM	27	0.44	27	0.18	0.26	(0.16, 0.36)
	PM	27	0.44	27	0.07	0.38	(0.28, 0.48)
1	AM	27	0.38	27	0.02	0.36	(0.26, 0.46)
	PM	27	0.41	27	0.15	0.26	(0.16, 0.36)
2	AM	27	0.37	27	0.03	0.34	(0.24, 0.44)
	PM	27	0.41	27	0.16	0.24	(0.14, 0.34)
3	AM	27	0.31	27	0.05	0.26	(0.16, 0.36)
	PM	27	0.36	27	0.18	0.18	(0.08, 0.28)
4	AM	27	0.29	27	0.05	0.24	(0.14, 0.34)
	PM	27	0.34	26	0.18	0.17	(0.07, 0.27)
5	AM	27	0.24	26	0.17	0.07	(−0.03, 0.17)
7^a^	AM	27	0.13	24	0.09	0.04	(−0.06, 0.14)
21^a^	AM	27	0.06	27	0.03	0.03	(−0.07, 0.13)

Legend: Summary of repeated measures statistical analysis of change from baseline forced expiratory volume in one second (FEV_1_), in litres, after cessation of repeat dose of treatment. Day 1 to Day 5 data was collected whilst subjects were in-patients. ^a^Day 7 and Day 21 measurements were taken during outpatient visits

Blood eosinophil and serum periostin levels throughout the study are shown in Table 4. Baseline blood eosinophils were ≥ 0.27 × 10^9/L for 24 participants (88.9%), but the majority of participants had blood eosinophil levels within the normal range throughout the study. Blood eosinophils were reduced after 14 days treatment with FF/VI 100/25 mcg; percentage change from baseline, FF/VI minus placebo, (95%CI), − 17.9% (− 33.2 to − 2.64). The point estimate for serum periostin level change from baseline was lower after FF/VI treatment, however the confidence interval included zero treatment difference, − 6.3% (− 18.5 to 5.9).

**Table 4:** Blood eosinophil and serum periostin levels throughout the study, by intervention

Day	Eosinophils ×10^9/L			Periostin ng/ml		
	FF/VI Median (min,max)	Placebo Median (min,max)	Point estimate of change from baseline (FF/VI – placebo)	FF/VI Median (min,max)	Placebo Median (min,max)	Point estimate of change from baseline (FF/VI – placebo)
Pre-dose	0.35 (0.20, 1.07)	0.34 (0.18, 1.18)	–	191.10 (109.40, 442.50)	189.10 (99.40, 398.60)	–
After 14 days of treatment (day 15)	0.30 (0.18, 0.86)	0.38 (0.17, 0.99)	−0.06 (− 0.14, 0.02)	150.00 (84.10, 823.40)	171.10 (87.80, 386.40)	−3.56 (−44.36, 37.24)
Seven days after cessation of treatment (day 21)	0.30 (0.04, 0.85)	0.29 (0.13, 0.70)	−0.02 (− 0.1, 0.08)	173.50 (94.90, 471.00)	175.70 (102.10, 389.90)	−8.86 (−30.66, 12.94)
21 days after cessation of treatment (Day 35)	0.36 (0.17, 0.87)	0.40 (0.21, 1.09)	−0.04 (− 0.12, 0.06)	171.70 (100.80, 457.70)	185.90 (113.30, 359.60)	−5.34 (−30.16, 19.46)

No serious adverse events were reported during the study. Seventeen (63%) subjects reported adverse events during the study (Additional file 1: Table S3). The most frequently reported adverse events in the study were viral upper respiratory tract infection (reported by 10 subjects, 37%); and back pain, musculoskeletal pain, and epistaxis (reported by 2 subjects each, 7%). Only one adverse event, cough with moderate intensity, was reported as potentially drug related. This resolved prior to cessation of study medication.

## Discussion

In this placebo-controlled study of the time-course of anti-inflammatory activity of FF/VI in asthma, treatment with FF/VI 100/25 mcg for 14 days led to the suppression of FeNO for approximately 18 days after withdrawal of treatment. In contrast, following cessation of FF/VI treatment, significant bronchodilation persisted for three to four days. This indicates that FF/VI exhibits a prolonged anti-inflammatory effect consistent with the high glucocorticoid receptor affinity and long lung retention of fluticasone furoate. The anti-inflammatory activity of FF/VI lasts longer than the bronchodilator effect, and therefore unopposed bronchodilator activity is unlikely to occur with FF/VI, a finding relevant to the scenario where patients omit one or more doses.

There are a number of methodological issues pertinent to the interpretation of the study findings. FeNO was used as the primary measure to monitor the anti-inflammatory activity of FF/VI as it is a validated biomarker of Type 2 inflammation in asthma, highly sensitive to ICS therapy, and, unlike alternative biomakers such as sputum or blood eosinophils or serum periostin, can be repeated twice daily in both a home and inpatient setting [11]. To ensure that the anti-inflammatory activity of FF/VI could be detected using FeNO, a screening FeNO > 40 ppb was used as an inclusion criterion. A FeNO value above 40 ppb predicts responsiveness to ICS in patients with non-specific airways disease, and is the recommended cut-off denoting a ‘high’ FeNO in the National Institute for Clinical Excellence (NICE) asthma guidelines [12, 13]. As FeNO is suppressed by ICS treatment and cigarette smoking, participants were required to be steroid naïve and not current smokers at baseline. We advised participants to follow a low nitrate diet throughout the study, and strictly ensured this was the case during their inpatient stay. The fall in FeNO in both groups towards the end of the inpatient period may be due to a combination of enforcement of the low nitrate diet and reduced exposure to allergens and environmental pollutants. A 21-day washout period was employed during which time participants were only allowed to take their SABA, with the aim of ensuring that the effect of FF/VI on FeNO had fully resolved. The prolonged time-course of anti-inflammatory activity seen in this study means that for subjects who received FF/VI in period 1 their pre-dose baseline for period 2 was approximately 11% lower than the period 1 pre-dose value. For subjects who received placebo in period 1 their pre-dose baseline for period 2 was approximately 2% lower than the period 1 pre-dose value (Additional file 1: Table S2). However, change in FeNO was assessed relative to the pre-dose value at the start of each treatment period, and in the statistical model used to estimate the primary endpoint “period” was included as a term and shown not to be a significant factor, confirming that there was no significant treatment carry-over between periods. Therefore any trend for a lower FeNO value at the start of period 2 was small and not a confounding factor in the study or its conclusions.

Participants were required to demonstrate a minimum FEV_1_ of at least 60% predicted as a safety criterion, and at least 12% bronchodilator reversibility at baseline in order to confirm the diagnosis of asthma. The requirement for a steroid naïve population who were able to tolerate ICS treatment being withheld for up to 21 days required us to recruit only participants with mild asthma. This, in addition to the exclusion of smokers and patients with FeNO levels < 40 ppb, mean that these results may not be generalizable to a wider asthmatic population. Participants were required to remain as inpatients for five days after cessation of treatment to ensure that their environment was as tightly controlled as possible, to avoid exposures that may influence FeNO or lung function measurements. This also allowed us to measure FEV_1_, which is a more sensitive measure of airflow obstruction than PEF and is a recommended core outcome measure in clinical trials [14].

It was not possible to determine the relative contributions of FF and VI to the anti-inflammatory and bronchodilator actions of combination FF/VI. However, based on the known properties of FF and VI demonstrated in the clinical trial program that lead to the registration of FF/VI [15], and previous reports that short-acting beta-agonists and LABAs do not affect FeNO [16, 17], the anti-inflammatory activity is likely to be due to the FF component.

FF/VI reduced FeNO within three days of the onset of treatment, with a progressive reduction to about one third of the baseline value by day 14. This is similar to the time-course of onset of FeNO suppression previously reported for inhaled budesonide, fluticasone propionate (FP), ciclesonide, and combination extra fine beclomethasone dipropionate/formoterol [18–21]. In contrast the suppression of FeNO persisted for 18 days after cessation of FF/VI treatment, which is appreciably longer than that observed with other ICS. Following treatment with budesonide (100mcg or 400mcg once-daily) [20] for three weeks, the FeNO returned to baseline levels by the end of a one week washout period. Following two weeks of treatment with beclomethasone diproprionate (200mcg/day) the FeNO returned to baseline within one to two weeks in the majority of participants [22]. Similarly, following FP (500mcg twice-daily) [19] the FeNO returned to baseline after two weeks, although no earlier time point measurements were included.

In addition to the changes seen with FeNO, two weeks of treatment with FF/VI reduced the blood eosinophil count by 17%. This finding is in keeping with the limited data available on the effect of ICS on blood eosinophils. In two separate studies in people with moderate asthma, two weeks of FP / salmeterol 100mcg/50mcg twice daily [23] and six weeks of budesonide 800mcg per day [24] were associated with reductions in blood eosinophils of 23 and 26% respectively. The reduction in blood eosinophils with ICS treatment is likely to be associated with a reduction in sputum eosinophils, as blood eosinophils are very strongly correlated with sputum eosinophils [23]. Blood eosinophil levels, although within the normal range for the majority of participants, were ≥ 0.27 × 10^9/L at baseline for 24 (88.9%). This threshold has previously been shown to have a 78% sensitivity and 91% specificity for sputum eosinophilia in participants with mild to moderate asthma [25], so both baseline FeNO and blood eosinophil data are consistent with participants in this study having eosinophilic airway inflammation at baseline. Periostin is associated with a type 2 pattern of inflammation [26], the sensitivity and specificity of the assay used in this study for the prediction of airway eosinophilia has not yet been determined. The point estimate of a 6% fall in periostin is in keeping with prior published data, with 5 and 12% reductions in periostin reported with FP 400mcg daily [27] and budesonide 800mcg daily [28] respectively.

It is difficult to compare the FeNO findings with other ICS as different equivalent daily doses were investigated, which confounds the assessment of responsiveness, as both the onset and offset of ICS-induced reduction in FeNO may be dose dependent [20, 29–31]. In addition, the baseline FeNO is a determinant of the magnitude and speed of ICS response [20], and the pre-treatment FeNO levels and sampling methodologies were markedly different between studies, with a range of between 6.9 ppb [19] and 122 ppb [32], compared with the baseline value of 87 ppb observed in this study. It is notable that for FF the offset of FeNO suppression was slower than the onset of action. This differs from the pattern previously reported with budesonide where, although the rate of onset and offset of action differed between the 100mcg and 400mcg doses, the rates of onset and offset were similar at each dose [20].

The ICS/LABA combination product studied in this investigation contains two molecules that both contribute to its long duration of action. Compared to other ICS, fluticasone furoate exhibits higher binding affinity and a longer duration of occupancy of the ligand binding domain of the glucocorticoid receptor [7]. The structural features of the FF molecule also confer physicochemical properties that result in higher affinity for respiratory tissue and longer lung retention compared to other ICS. Through in vitro and in vivo studies, FF has been demonstrated to have a longer intracellular duration of action than FP [33], as well as higher potency [33], prolonged intracellular retention [7] and prolonged lung absorption kinetics [7, 8]. Vilanterol has a high β_2_ receptor affinity, with rapid onset of action and relatively slow dissociation [34]. The prolonged bronchodilator action of vilanterol may be due primarily to it being a highly lipophilic molecule, leading to the formation of ‘depots’ in the cell membrane resulting in persistence and reassertion at the β_2_-receptor [34].

This prolonged anti-inflammatory effect of FF/VI may have implications for poorly adherent patients as it confirms that the anti-inflammatory activity works alongside the bronchodilator activity for the approved once-daily dosing regimen, and also suggests that patients who miss one or more doses of FF/VI will still receive both anti-inflammatory and bronchodilator coverage for some time after the last dose. Finally, it provides reassurance that they will not be exposed to unopposed LABA activity as they are likely to still derive benefits from its anti-inflammatory effect. However, dosing less frequently than once a day has not been studied and FF/VI is only licensed for use as a once-daily medication.

## Conclusions

Among adult patients with asthma who were not taking ICS at baseline, treatment with FF/VI at a dose of 100/25mcg once daily for 14 days substantially reduced airway inflammation, as measured by FeNO suppression. After cessation of treatment, FeNO suppression persisted for approximately 18 days, whereas clinically significant bronchodilation persisted for three to four days. These results show that the duration of anti-inflammatory action of FF/VI is longer than has previously been reported for other ICS studied and exceeds the duration of bronchodilation. Therefore, even when adherence to therapy is poor, there is prolonged anti-inflammatory activity and no unopposed LABA activity with this combination ICS/LABA.

## Additional file

Additional file 1: **Supplementary appendix:** The anti-inflammatory duration of action of fluticasone furoate/vilanterol trifenitate in asthma. (DOCX 97 kb)

## Abbreviations

FeNO: Fraction of exhaled nitric oxide
FF: Fluticasone furoate
ICS: Inhaled corticosteroids
LABA: Long-acting beta-agonists
VI: Vilanterol
