# Supplementary material for: Anti-inflammatory duration of action of fluticasone furoate/vilanterol trifenatate in asthma: a cross-over randomised controlled trial
Source: Respir Res. 2018 Jul 13;19:133. doi: 10.1186/s12931-018-0836-6 (PMC6044077; doi:10.1186/s12931-018-0836-6)
Supplement: Supplementary file 1 — Supplementary appendix: The anti-inflammatory duration of action of fluticasone furoate/vilanterol trifenitate in asthma. (DOCX 97 kb) [file 12931_2018_836_MOESM1_ESM.docx]

Additional file 1: The anti-inflammatory duration of action of fluticasone furoate/vilanterol trifenitate in asthma

George Bardsley[^1^](http://www.sciencedirect.com/science/article/pii/S0954611116302293#aff1), Peter Daley-Yates[^2^](http://www.sciencedirect.com/science/article/pii/S0954611116302293#aff1), Amanda Baines[^3^](http://www.sciencedirect.com/science/article/pii/S0954611116302293#aff1), Rodger Kempsford^3^ Mathew Williams[^1^](http://www.sciencedirect.com/science/article/pii/S0954611116302293#aff1), Tony Mallon^1^, Irene Braithwaite^1^, Kylie Riddell[^4^](http://www.sciencedirect.com/science/article/pii/S0954611116302293#aff1), Shashidhar Joshi[^5^](http://www.sciencedirect.com/science/article/pii/S0954611116302293#aff1), Philippe Bareille[^3^](http://www.sciencedirect.com/science/article/pii/S0954611116302293#aff1), Richard Beasley[^1^](http://www.sciencedirect.com/science/article/pii/S0954611116302293#aff1), and James Fingleton[^1^](http://www.sciencedirect.com/science/article/pii/S0954611116302293#aff1) on behalf of the study team

^1^ Medical Research Institute of New Zealand, Wellington,

^2^ Respiratory Clinical Development, GlaxoSmithKline Research and Development, Stockley Park, UK

^3^ Medicines Development Centre, GlaxoSmithKline Research and Development, Stevenage, UK

^4^ GlaxoSmithKline Research and Development, 82 Hughes Ave, Ermington NSW 2115, Australia

^5^ Quantitative sciences, GlaxoSmithKline Bangalore, India

**Contact:**

Dr James Fingleton

Medical Research Institute of New Zealand

Private Bag 7902, Newtown

Wellington 6242, New Zealand

Telephone: +64-4-805 0247

Facsimile: +64-4-389 5707

Email: [james.fingleton@mrinz.ac.nz](mailto:james.fingleton@mrinz.ac.nz)

**Total word count** (max 4500): 4,100

**Figure S1:** Screening, Randomization, and Follow-up of patients

###

### Periostin analysis methodology

An abbreviated validation of a method for the determination of the representative peptide

‘NGAIHIFR’ for Periostin in human serum (using 4% Bovine Serum Albumin (BSA) in

Phosphate Buffered Saline (PBS) as the surrogate matrix) has been validated over the

range 10 to 500 ng/mL using immunocapture UHPLC-MS/MS. Periostin was

immunocaptured using a biotinylated mouse anti-human Periostin antibody from 50 μL

of human serum or surrogate matrix diluted 4-fold in assay buffer. Immunocaptured

samples are tryptically digested in the presence of an internal standard

([H]NGAIHI[^13^C_9_^15^N-F]R [OH]). Digested samples were analyzed by UHPLC-MS/MS

using a TurboIonSpray™ interface and multiple reaction monitoring. The method is

selective for the peptide ‘NGAIHIFR’, and a summary of the validation data is tabulated

below.

| Calibration Model | Linear - Weighted 1/(x*x) |
| --- | --- |
| Validated Range | 10 – 500 ng/mL |
| Precision (%CV)  Within-run | ≤ 26.7% for surrogate matrix  ≤ 11.7% for human serum |
| Precision (%CV)  Between-run | ≤ 15.4% for surrogate matrix  ≤ 9.4% for human serum |
| Accuracy (%bias)  Within-run | 4.8% ≤ Bias ≤ 29.4% for surrogate matrix  4.4% ≤ Bias ≤ -3.9% for human serum |
| Total Error (%)  Within-run | ≤ 38.0% for surrogate matrix (LLQ)  ≤ 16.1% for human serum |
| Total Error (%)  Between-run | ≤ 29.6% for surrogate matrix (LLQ)  ≤ 9.4% for human serum |
| Freeze-Thaw Stability in Human Serum | 3 cycles from -80°C to ambient temperature |
| Stability in Human Serum | 24 hours at ambient temperature |
| Human Serum Dilution in 4% BSA in PBS | 4- Fold in 4% BSA in PBS (surrogate matrix) |

## Inclusion Criteria

A subject will be eligible for inclusion in this study only if all of the following criteria apply:

| AGE |
| --- |
| 1. **Age of subject**: Between 18 and 65 years of age inclusive, at the time of signing the informed consent. |

| TYPE OF SUBJECT AND DIAGNOSIS INCLUDING DISEASE SEVERITY |
| --- |
| 1. **Asthma**: A doctor diagnosis of asthma for at least 6 months prior to the start of the study. 2. **Severity of disease**: A screening pre-bronchodilator FEV_1_ ≥ 60% of predicted |

| NOTE: Predicted values will be based upon NHANES III [[Hankinson](#_bookmark31), 1999]   1. **Reversibility of disease**:   Demonstrated presence of reversible airway disease at screening (repeat testing of eligibility can be undertaken following the screening visit up to Day -7).  OR  The presence of reversible airways disease can have been demonstrated historically within 6 months of the screening visit.  NOTE: Reversible airway disease is defined as increase in FEV_1_ of ≥ 12% over baseline and an absolute change of ≥ 200 mL within 30 minutes following 4 inhalations of albuterol/salbutamol inhalation aerosol/spacer (or equivalent nebulised treatment with albuterol/salbutamol solution).   1. **Current Therapy:**    - Short-Acting Beta2-Agonists (SABA): prescribed SABA for at least 12 weeks prior to screening.   No ICS, LABA, long acting muscarinic anatagonist (LAMA), leukotriene receptor antagonist (LTRA) therapy for three months prior to the start of the study.   1. Non-smoker or ex-smoker (no smoking in previous 12 weeks, ≤10 pack years). 2. Screening and Day -7 AM FeNO values > 40ppb.   NOTE: Both screening and Day -7 AM FeNO values for Treatment Period 1 need to be > 40ppb for the subject to be eligible. |
| --- |

| WEIGHT |
| --- |
| 8. **Bodyweight and BMI:** Bodyweight ≥ 50 kg and Body Mass Index (BMI) within the range 18.0-40.0 kg/m^2^ (inclusive) |

| SEX |
| --- |
| 1. Male OR Female   **Females**:  A female subject is eligible to participate if she is not pregnant (as confirmed by a negative urine human chorionic gonadotrophin (hCG) test), not lactating, and at least one of the following conditions applies:Non-reproductive potential defined as:   - - Pre-menopausal females reporting one of the following:     - Tubal ligation     - Hysteroscopic tubal occlusion procedure with follow-up confirmation of bilateral tubal occlusion     - Hysterectomy |

| - Bilateral Oophorectomy - Postmenopausal defined as 12 months of spontaneous amenorrhea [in questionable cases a blood sample with simultaneous follicle stimulating hormone (FSH) and estradiol levels consistent with menopause (refer to laboratory reference ranges for confirmatory levels)]. Females on hormone replacement therapy (HRT) and whose menopausal status is in doubt will be required to use one of the highly effective contraception methods if they wish to continue their HRT during the study. Otherwise, they must discontinue HRT to allow confirmation of post-menopausal status prior to study enrolment.   b. Reproductive potential and agrees to follow one of the options listed in the Modified List of Highly Effective Methods for Avoiding Pregnancy in Females of Reproductive Potential (FRP) (see Appendix 3) from 30 days prior to the first dose of study medication and until after the last dose of study medication and completion of the follow-up visit.  The investigator is responsible for ensuring that subjects understand how to properly use these methods of contraception. |
| --- |

| INFORMED CONSENT |
| --- |
| 10. Capable of giving signed informed consent, which includes compliance with the requirements and restrictions listed in the consent form and in this protocol. |

## Exclusion Criteria

Subjects were not be eligible for inclusion in this study if any of the following criteria applied:

| CONCURRENT CONDITIONS/MEDICAL HISTORY |
| --- |
| 1. **A history of life-threatening asthma**.   NOTE**:** Life-threatening asthma is defined as an asthma episode that required intubation and/or was associated with hypercapnia, respiratory arrest or hypoxic seizures within the last 5 years   1. **Other significant pulmonary diseases to include (but not limited to):** pneumonia, pneumothorax, atelectasis, pulmonary fibrotic disease, bronchopulmonary dysplasia, chronic bronchitis, emphysema, chronic obstructive pulmonary disease, or other respiratory abnormalities other than asthma. 2. **Respiratory Infection:** Culture-documented or suspected bacterial or viral infection of the upper or lower respiratory tract, sinus or middle ear that is not resolved within 4 weeks of screening that:  - led to a change in asthma management OR - in the opinion of the Investigator, is expected to affect the subject’s asthma status OR |

| the subject’s ability to participate in the study.  However, subjects can be rescreened to allow for an adequate time period (of at least 4 weeks) between resolution of the infection and the date of randomisation.  **4. Asthma Exacerbation:** Any asthma exacerbation requiring oral corticosteroids within 12 weeks of screening or that resulted in overnight hospitalization requiring additional treatment for asthma within 6 months prior to screening. |
| --- |

| CONCOMITANT MEDICATIONS |
| --- |
| **5. Concomitant Medications:** These are listed in detail in Section 6.9 of the protocol. Restrictions of nitrate-rich foods are listed in detail in Section 6.9.1.1 of the protocol. |

| RELEVANT HABITS |
| --- |
| **6. Tobacco Use:** Current smokers or a smoking history of ≥ 10 pack years. A subject may not have used any inhaled tobacco products in 12 weeks preceding the screening visit. |

| CONTRAINDICATIONS |
| --- |
| **7. Previous Participation:** Exposure to more than four new chemical entities within 12 months prior to the first dosing day. |

| DIAGNOSTIC ASSESSMENTS AND OTHER CRITERIA | | | |
| --- | --- | --- | --- |
| **8. Other concurrent Diseases/Abnormalities:** A subject has any clinically significant, uncontrolled condition or disease state that, in the opinion of the investigator, would put the safety of the subject at risk through study participation or would confound the interpretation of the study results if the condition/disease exacerbated during the study.  The list of additional excluded conditions/diseases includes, but is not limited to the following: | | | |
|  | Congestive heart failure | Known aortic aneurysm |  |
|  | Clinically significant coronary heart disease | Clinically significant cardiac arrhythmia |  |
|  | Stroke within 3 months of Visit 1 | Uncontrolled hypertension* |  |
|  | Recent or poorly controlled peptic ulcer | Haematologic, hepatic**, or renal disease |  |
|  | Immunologic compromise | Current malignancy*** |  |
|  | Tuberculosis (current or untreated)**** | Cushing’s disease |  |
|  | Addison’s disease | Uncontrolled diabetes mellitus |  |
|  | Liver cirrhosis | Systemic Lupus Erythematosus |  |
|  | Uncontrolled thyroid disorder | Recent history of drug or alcohol abuse |  |

| *Two or more measurements with systolic BP>160mmHg, or diastolic BP >100mmHg  **Subjects with chronic stable hepatitis B and C are acceptable provided their screening ALT is < 2x upper limit of normal (ULN) and the subject otherwise meets the entry criteria. Subjects who have chronic co-infection with both hepatitis B and hepatitis C are not eligible.  ***history of malignancy is acceptable only if subject has been in remission for one year prior to Visit 1 (remission = no current evidence of malignancy and no treatment for the malignancy in the 12 months prior to Visit 1)  ****Subjects with a history of tuberculosis infection who have completed an appropriate course of antituberculous treatment may be suitable for study entry provided that there is no clinical suspicion of active or recurrent disease.   1. **Oropharyngeal examination**: A subject will not be eligible if he/she has clinical visual evidence of oral candidiasis at screening. 2. **Pregnancy and Lactating Females**:    - Pregnant females as determined by positive serum hCG test at screening or by positive urine hCG test prior to dosing.    - Lactating females 3. **Allergies:**    - **Milk Protein Allergy:** History of severe milk protein allergy.    - **Drug Allergy:** Any adverse reaction including immediate or delayed hypersensitivity to any beta_2_-agonist, sympathomimetic drug, or any intranasal, inhaled, or systemic corticosteroid therapy. Known or suspected sensitivity to the constituents of the Dry Powder Inhaler (DPI) (i.e., lactose or magnesium stearate).    - **Historical Allergy:** History of drug or other allergy that, in the opinion of the investigator or GSK Medical Monitor, contraindicates their participation. |
| --- |

### Table S1: Summary of Repeated Measures Statistical Analysis of Change from Baseline FEV1 (L) Data

| **Day Relative to Last dose** | **Planned Time point** | **Adjusted Mean** | | | | **Difference (FF/VI 100/25 mcg vs Placebo)** | **95% CI of the Difference** |
| --- | --- | --- | --- | --- | --- | --- | --- |
|  |  | **n** | **FF/VI 100/25 mcg** | **n** | **Placebo** |  |  |
| 0 | AM assessment | 27 | 0.44 | 27 | 0.18 | 0.26 | (0.16, 0.36) |
|  | PM assessment | 27 | 0.44 | 27 | 0.07 | 0.38 | (0.28, 0.48) |
| 1 | AM assessment | 27 | 0.38 | 27 | 0.02 | 0.36 | (0.26, 0.46) |
|  | PM assessment | 27 | 0.41 | 27 | 0.15 | 0.26 | (0.16, 0.36) |
| 2 | AM assessment | 27 | 0.37 | 27 | 0.03 | 0.34 | (0.24, 0.44) |
|  | PM assessment | 27 | 0.41 | 27 | 0.16 | 0.24 | (0.14, 0.34) |
| 3 | AM assessment | 27 | 0.31 | 27 | 0.05 | 0.26 | (0.16, 0.36) |
|  | PM assessment | 27 | 0.36 | 27 | 0.18 | 0.18 | (0.08, 0.28) |
| 4 | AM assessment | 27 | 0.29 | 27 | 0.05 | 0.24 | (0.14, 0.34) |
|  | PM assessment | 27 | 0.34 | 26 | 0.18 | 0.17 | (0.07, 0.27) |
| 5 | AM assessment | 27 | 0.24 | 26 | 0.17 | 0.07 | (-0.03, 0.17) |
| 7* | AM assessment | 27 | 0.13 | 24 | 0.09 | 0.04 | (-0.06, 0.14) |
| 21* | AM assessment | 27 | 0.06 | 27 | 0.03 | 0.03 | (-0.07, 0.13) |
| *****Day 1 to Day 5 data was collected whilst subjects were in-patients. Day 7 and Day 21 measurements were taken during outpatient visits. | | | | | | | |

### Table S2: Summary FeNO pre-dose baseline data by treatment sequence and period.

| **Period 1** | ***FeNO ppb** | **Period 2** | ***FeNO ppb** |
| --- | --- | --- | --- |
| FF/VI | 74.5 (57.3-96.9) | Placebo | 65.9 (50.6-85.8) |
| Placebo | 104.0 (79.3-136.5) | FF/VI | 102.1 (72.0-144.9) |

*Geometric mean and CI

## Adverse events

Seventeen (63%) subjects reported AEs during the study (Table S2). A total of 42 AEs were experienced across a total of 27 subjects. The most frequently reported AEs in the study were viral upper respiratory tract infection (reported by 10 subjects, 37%) and back pain, musculoskeletal pain, epistaxis (reported by 2 subjects each, 7%). All other AEs were reported by 1 subject. Viral pharyngitis, musculoskeletal pain, neck pain, pain in extremity, epistaxis, cough, gingival pain, nausea, influenza like illness, vessel puncture site pain, insomnia and rash were reported in FF/VI 100/25 mcg group only. Orchitis, urinary tract infection, back pain, oropharyngeal pain, food poisoning and food allergy were reported in placebo group only.

Table S3 Summary of All Adverse Events by Treatment (Safety Population)

| **Preferred Term** | **Placebo (N=27)** | **FF/VI 100/25 mcg (N=27)** | **Overall (N=27)** |
| --- | --- | --- | --- |
| **Any Event, n (%)** | 10 (37) | 14 (52) | 17 (63) |
| Viral upper respiratory tract infection | 5 (19) | 7 (26) | 10 (37) |
| Back pain | 2 (7) | 0 | 2 (7) |
| Epistaxis | 0 | 2 (7) | 2 (7) |
| Musculoskeletal pain | 0 | 2 (7) | 2 (7) |
| Cough | 0 | 1 (4) | 1 (4) |
| Food poisoning | 1 (4) | 0 | 1 (4) |
| Food allergy | 1 (4) | 0 | 1 (4) |
| Gingival pain | 0 | 1 (4) | 1 (4) |
| Insomnia | 0 | 1 (4) | 1 (4) |
| Influenza like illness | 0 | 1(4) | 1(4) |
| Nausea | 0 | 1 (4) | 1 (4) |
| Neck pain | 0 | 1 (4) | 1 (4) |
| Orchitis | 1 (4) | 0 | 1 (4) |
| Oropharyngeal pain | 1 (4) | 0 | 1 (4) |
| Pain in extremity | 0 | 1 (4) | 1 (4) |
| Rash | 0 | 1 (4) | 1 (4) |
| Urinary tract infection | 1 (4) | 0 | 1 (4) |
| Viral pharyngitis | 0 | 1 (4) | 1 (4) |
| Vessel puncture site pain | 0 | 1 (4) | 1 (4) |
|  | | | |
